# Placental galectin-3 is reduced in early-onset preeclampsia

**DOI:** 10.3389/fphys.2022.1037597

**Published:** 2022-10-14

**Authors:** Manju Kandel, Stephen Tong, Susan P Walker, Ping Cannon, Tuong-Vi Nguyen, Teresa M. MacDonald, Natalie J. Hannan, Tu’uhevaha J. Kaitu’u-Lino, Lucy A Bartho

**Affiliations:** ^1^ Translational Obstetrics Group, The Department of Obstetrics and Gynaecology, Mercy Hospital for Women, University of Melbourne, Heidelberg, VIC, Australia; ^2^ Mercy Perinatal, Mercy Hospital for Women, Heidelberg, VIC, Australia

**Keywords:** placenta, preeclampsia, galectin-3, galectin-3 binding protein, plasma

## Abstract

Preeclampsia is a disease of pregnancy responsible for significant maternal and neonatal mortality. Galectin-3 is a *β*-Galactoside binding protein. This study aimed to characterise galectin-3 in women with preeclampsia and human trophoblast stem cells (hTSCs). Galectin-3 was measured in placental lysates and plasma collected from patients with early-onset preeclampsia (delivered <34 weeks’ gestation) and gestation matched controls. Placental galectin-3 protein was significantly reduced in 43 women with early-onset preeclampsia compared to 21 controls. mRNA expression of *LGALS3* (galectin-3 encoding gene) was reduced in 29 women with early-onset preeclampsia, compared to 18 controls (*p* = 0.009). There was no significant difference in plasma galectin-3 protein in 46 women with early-onset preeclampsia compared to 20 controls. In a separate cohort of samples collected at 36 weeks’ gestation, circulating galectin-3 was not altered in 23 women who later developed preeclampsia, versus 182 who did not. In syncytialised hTSCs, hypoxia increased mRNA expression of *LGALS3* (*p* = 0.01). Treatment with inflammatory cytokines (TNF-α and IL-6) had no effect on *LGALS3* mRNA expression*.* However, TNF-α treatment caused an increase in mRNA expression of *LGALS3BP* (galectin-3 binding protein encoding gene) in hTSCs (*p* = 0.03). This study showed a reduction of galectin-3 in placenta from pregnancies complicated by early-onset preeclampsia. *LGALS3* mRNA expression was dysregulated by hypoxia exposure in placental stem cells.

## 1 Introduction

Preeclampsia is a pregnancy-specific disorder which affects 2%–8% of mothers (Groot et al., 2016). It is characterised by placental hypoxia, local and systemic inflammation (Roberts and Gammill, 2005).

Galectins are a family of *β*-Galactoside binding proteins important for successful implantation and maintenance of pregnancy (Jovanović Krivokuća et al., 2021). Galectin-3 (encoded by the *LGALS3* gene) is abundantly expressed at the maternal-fetal interface and secreted from the cell surface into biological fluids (Blois et al., 2015; Blois et al., 2007; Than et al., 2014; Tirado-Gonzalez et al., 2012). Galectin-3 binding protein (galectin-3BP), encoded by the *LGALS3BP* gene, is a highly glycosylated protein that acts as a ligand for several galectins, including galectin-3 (Lin et al., 2015). Galectin-3 regulates various cellular processes including cellular growth, differentiation, and inflammation (Liu et al., 2002). Dysregulated galectin-3 has been characterised in the pathogenesis of several diseases, such as heart failure, cancer and pulmonary hypertension (Sciacchitano et al., 2018).

In placenta, galectin-3 is expressed in all trophoblastic lineages including cytotrophoblasts and extravillous trophoblasts (Maquoi et al., 1997; Vićovac et al., 1998) and is released in response to hypoxia in BeWo choriocarcinoma cells (Hu et al., 2007). Several studies have shown that elevated galectin-3 levels are associated with preeclampsia (Amarilyo et al., 2011; Božić et al., 2004; Hu et al., 2007; Jeschke et al., 2007). Nikolov et al. (2020) found no change in serum galectin-3 levels between patients with preeclampsia compared to control. These results are likely due to underpowered sample numbers, hence the conflicted results.

Therefore, this study aimed to assess galectin-3 mRNA expression and protein levels in placentas and plasma using two well-defined cohorts, including one with confirmed early-onset preeclampsia, and another collected prior to diagnosis of term preeclampsia. Additionally, we assessed galectin-3 and galectin-3BP levels in an *in vitro* model of preeclampsia where differentiated human trophoblast stem cells (hTSCs) (Okae et al., 2018) were exposed to hypoxia and pro-inflammatory cytokines.

## 2 Materials and methods

### 2.1 Placenta and plasma collection at less than 34 weeks’ gestation

Ethics approval was obtained from Mercy Health Human Research Ethics Committee (R11/34). Patients presenting to Mercy Hospital for Women (Heidelberg, Victoria) gave written, informed consent for collection of blood during their pregnancy, and placentas following caesarean delivery. Placentas were obtained from patients with established early-onset preeclampsia (<34 weeks’ gestation; *n* = 43) and gestation matched controls (*n* = 21). Placentas were processed within 30 min of delivery where tissue was sampled from four quadrants of the placenta and washed in phosphate buffered saline. Placental tissue was processed with RNA*later*™ stabilization solution and stored at −80°C for future analysis. Preeclampsia was diagnosed in accordance with the American College of Obstetrics and Gynaecology (ACOG) guidelines (2020) (Obstetricians ACo Gynecologists, 2020). Refer to Table 1 and Table 2 for patient characteristics.

**TABLE 1:** Maternal characteristics and pregnancy outcomes for less than 34 weeks placental samples—measured galectin-3 protein.

	Controls (*n* = 21)	Preeclampsia (*n* = 43)	*p*-value
Maternal age (years)	30.14 ± 1.61	31.51 ± 0.83	0.41
Mean ± SEM			
Gestation at delivery (weeks)	30.11 ± 0.54	30.20 ± 0.36	0.89
Mean ± SEM			
Body mass index (kg/m^2^)	28.20 (24.25–34.85)	27.00 (25.00–35.60)	0.99
Median (IQR)			
Parity no. (%)			
0	5 (23.81)	32 (74.42)	0.0006
1	11 (52.38)	7 (16.28)	
≥2	5 (23.81)	4 (9.30)	
SBP at delivery (mmHg)	120 (111–130)	170 (160–185)	<0.0001
Median (IQR)			
DBP at delivery (mmHg)	70 (67.50–80.00)	100 (95.00–110.00)	<0.0001
Median (IQR)			
Birthweight (g)	1,585 (1,278–1,943)	1,253 (866.8–1,479)	0.01
Median (IQR)			
Male no. (%)	11 (52.38)	23 (53.49)	0.93

BMI, body mass index; SBP, systolic blood pressure; DBP, diastolic blood pressure. Unpaired t-test was used for normally distributed data, Mann-Whitney U tests for non-parametric data, and Chi-square tests for categorical variables. BMI data missing for 5/21 control samples, and 10/43 preeclampsia samples. Birthweight data missing for 1/43 PE samples. *p* < 0.05 was considered significant.

**TABLE 2:** Maternal characteristics and pregnancy outcomes for less than 34 weeks placental samples—measured galectin-3 mRNA expression.

	Controls (*n* = 18)	Preeclampsia (*n* = 29)	*p*-value
Maternal age (years)	31.50 ± 1.64	32.52 ± 0.95	0.56
Mean ± SEM			
Gestation at delivery (weeks)	30.25 ± 0.59	31.12 ± 0.24	0.13
Mean ± SEM			
BMI (kg/m^2^)	28.20 (24.75–35.08)	26.80 (23.75–34.60)	0.34
Median (IQR)			
Parity no. (%)			
0	4 (22.2)	20 (68.97)	0.007
1	9 (50.0)	5 (17.24)	
≥2	5 (27.8)	4 (13.79)	
SBP at delivery (mmHg)	125 (117.3–130)	180 (169–183)	<0.0001
Median (IQR)			
DBP at delivery (mmHg)	75 (70.00–80.00)	100 (97.00–110.00)	<0.0001
Median (IQR)			
Birth weight (g)	1,587 (1,298–2,011)	1,425 (1,314–1,561)	0.22
Median (IQR)			
Male no. (%)	10 (55.56)	20 (68.97)	0.35

BMI, body mass index; SBP, systolic blood pressure; DBP, diastolic blood pressure. Unpaired t-test was used for normally distributed data, Mann-Whitney U tests for non-parametric data, and Chi-square tests for categorical variables. BMI data missing for 4/18 control samples, and 4/29 PE samples. *p* < 0.05 was considered significant.

Whole blood was collected in a 9 ml ethylenediaminetetraacetic acid (EDTA, BD Vacutainer® K2E) tube, centrifuged and plasma obtained from patients delivering at <34 weeks’ gestation with preeclampsia (*n* = 46), or gestation matched controls (*n* = 20) who delivered without preeclampsia at term. Plasma was stored at −80°C for further analysis. Refer to Table 3 for patient characteristics.

**TABLE 3:** Maternal Characteristics for less than <34 weeks plasma samples.

	Controls (*n* = 20)	Preeclampsia (*n* = 46)	*p*-value
Maternal age (years)	33.00 ± 1.103	30.83 ± 0.84	0.21
Mean ± SEM			
Gestation at blood collection (weeks)	27.61 ± 0.95	29.65 ± 0.39	0.07
Mean ± SEM			
Gestation at delivery (weeks)	39.06 ± 0.23	29.95 ± 0.43	<0.0001
Mean ± SEM			
Parity no. (%)			
0	8 (40.00)	33 (71.74)	0.04
1	7 (35.00)	9 (19.56)	
≥2	5 (25.00)	4 (8.70)	
SBP at delivery (mmHg)	120.00 (125.00–110.00)	173.50 (180.00–165.00)	<0.0001
Median (IQR)			
DBP at delivery (mmHg)	75.00 (80.00–70.00)	100.00 (110.00–99.75)	<0.0001
Median (IQR)			
BMI (kg/m^2^)	25.40 (30.05–20.40)	28.50 (35.20–26.00)	0.02
Median (IQR)			
Birth weight (g)	3,465 (3,873–3,180)	1,277 (1,625–777.50)	<0.0001
Median (IQR)			
Male no. (%)	8 (40.00)	19 (41.30)	0.92

BMI, body mass index; SBP, systolic blood pressure; DBP, diastolic blood pressure. Unpaired t-test was used for normally distributed data, Mann-Whitney U tests for non-parametric data, and Chi-square tests for categorical variables. BMI data missing for 3/46 PE samples; SBP and DBP data missing for 1/20 control samples. *p* < 0.05 was considered significant.

### 2.2 Plasma collected at 36 weeks’ gestation preceding preeclampsia diagnosis

The Biomarker and Ultrasound Measures for Preventable Stillbirth (BUMPS) study is a large prospective cohort conducted at the Mercy Hospital for Women (Heidelberg, Victoria). This cohort was designed to identify biomarkers for pregnancy complications. Ethics approval was obtained from the Mercy Health Research Committee (approval number: 2019–012). English speaking patients aged 18 years and over, with a singleton pregnancy and normal mid-trimester fetal morphology were eligible to participate. Patient whole blood were collected at 36 (35^+0^–37^+0^) weeks’ gestation in 9 ml EDTA vacutainers from 182 healthy controls and 23 patients who went on to develop term preeclampsia. Bloods were centrifuged and plasma was stored at −80°C until further analysis. Refer to Table 4 for patient characteristics.

**TABLE 4:** Patient characteristics and pregnancy outcomes for the Mercy Hospital for Women cohort who provided a blood sample at 36 weeks’ gestation.

	Controls (*n* = 182)	PE (*n* = 23)	*p*-value
Maternal age (years)	32.00 (29.75–35.00)	34.00 (32.00–37.00)	0.02
Median (IQR)			
Booking BMI (kg/m^2^)	24.55 (22.29–28.12)	27.88 (24.14–30.66)	0.02
Median (IQR)			
Parity no. (%)			
0	89 (48.90)	19 (82.61)	0.007
1	72 (39.56)	4 (17.39)	
≥2	21 (11.54)	0 (0.00)	
Smoking status no. (%)			
Current smoker	170 (93.40)	21 (91.30)	0.93
Ex-smoker	6 (3.30)	1 (4.35)	
Never smoked	6 (3.30)	1 (4.35)	
GDM no (%)	20 (11.11)	6 (26.10)	0.04
Onset of labour no. (%)			
Spontaneous	80 (43.96)	7 (30.43)	0.29
Induced	72 (39.56)	13 (56.52)	
No labour	30 (16.48)	3 (13.05)	
Caesarean section no. (%)	59 (32.42)	12 (52.17)	0.06
Gestation at delivery (weeks)	39.42 (38.85–40.42)	38.57 (37.85–39.28)	<0.0001
Median (IQR)			
Birth weight (g)	3,490 (3,185–3,820)	3,150 (2,550–3,450)	0.0002
Median (IQR)			
Male no. (%)	91 (50.00)	11 (47.83)	0.84

BMI, body mass index; GDM, gestational diabetes mellitus. Unpaired t-test was used for normally distributed data, Mann-Whitney U tests for non-parametric data, and Chi-square tests for categorical variables. BMI and GDM data missing for 2/182 control samples. *p* < 0.05 was considered significant.

### 2.3 Culture of first trimester human trophoblast stem cells

First trimester human trophoblast stem cell lines (hTSCs) were imported from the RIKEN BRC through the National BioResource Project of the MEXT/AMED, Japan. Cells were cultured according to the publication by Okae and colleagues (Okae et al., 2018).

#### 2.3.1 Differentiation of human trophoblast stem cells into syncytiotrophoblast or extravillous trophoblasts

First trimester cytotrophoblast stem cell lines (hTSCs) were differentiated into either syncytiotrophoblast or extravillous trophoblast cells (EVTs) as described previously (Okae et al., 2018).

#### 2.3.2 Treatment of syncytialised human trophoblast stem cells with interleukin 6, tumor necrosis factor α and hypoxia

Cells were plated at 60,000 cells/well in a 24-well cell culture plate in syncytial [ST(2D)] media and incubated at 37°C, 8% O_2_, and 5% CO_2_ for 72 h to allow for syncytialisation. Next, cells were incubated in a hypoxic environment or with inflammatory stimuli. Cells in a hypoxic environment were cultured at 1% O_2_ whilst normoxic cells were maintained at 8% O_2_ for an additional 48 h. To induce inflammation, cells were treated with increasing doses of tumor necrosis factor α (TNFα) or interleukin 6 (IL-6) at 0, 0.1, 1, and 10 ng/ml for 24 h. Experiments were treated in triplicates and repeated separately (*n* = 5).

### 2.4 Enzyme linked immunosorbent assay

Galectin-3 levels were measured in plasma and placenta protein lysate using human DuoSet ELISA kits (RnD systems; Catalogue # DY1154, Minnesota, United States) according to the manufacturer’s instructions.

### 2.5 RNA extraction, reverse transcription, and reverse transcriptase polymerase chain reaction

RNA was extracted from hTSCs with GenElute™ mammalian total RNA miniprep kit (Sigma-Aldrich) and quantified using a Nanodrop ND 1000 spectrophotometer (NanoDrop Technologies Inc., Wilmington, DE, United States). RNA was converted to cDNA with high-capacity cDNA reverse transcriptase kit (Applied Biosystems, Life Technologies) as per manufacturer’s instructions using iCycler iQ5 machine (Biorad) with run conditions: 25°C for 10 min, 37°C for 60 min and 85°C for 5 min.

Quantitative reverse transcriptase polymerase chain reaction (RT-PCR) measured the mRNA expression of genes; *LGALS3* (Assay ID: Hs00173587_m1), *LGALS3BP* (Assay ID: Hs00174774_m1), *TEAD4* (TEA Domain Transcription Factor 4, Assay ID: Hs01125032_m1), *SDC1* (Syndecan 1, Assay ID: Hs00896423_m1) and *HLAG* (Human Leukocyte Antigen G, Assay ID: Hs03045108_m1) using Fluorescein amidite (FAM) labelled Taqman gene expression assays (Life Technologies) on the CFX 384 (Biorad, Hercules, CA) with Taqman fast advanced universal PCR mastermix (Applied Biosystems). The run conditions were: 95°C for 20 s followed by 40 cycles of 95°C for 3 s and 60°C for 30 s. All data was normalized to the housekeeping gene *YWHAZ* (Tyrosine 3-Monooxygenase/Tryptophan 5-Monooxygenase Activation Protein Zeta*,* Assay ID: Hs01122454_m1) for inflammatory stimuli treated cells and the geometric mean of *TOP1* (topoisomerase‐1, Assay ID: Hs00243257_m1) or *CYC1* (cyclin‐1, Assay ID: Hs00357717_m1) for hypoxic and EVT cells. Samples were run in duplicate, and an average cycle threshold (Ct) value was used. Results were normalised to the Ct mean of each control group and expressed as a fold change with respect to the control.

### 2.6 Statistical analysis

Maternal characteristics were compared for patients with preeclampsia compared to controls using a Mann-Whitney U test for continuous data, and Chi-square test for categorical data. Data was initially assessed for normal distribution using Anderson-Darling test, D’Agostino and Pearson test, Shapiro-Wilk test, and Kolmogrov-Smirnov test. For data containing two groups, Mann-Whitney test was used for unpaired non-parametric data. For analysis comparing more than three groups, one-way analysis of variance (ANOVA; parametric) or Kruskal Wallis test (non-parametric) was used. *In vitro* experiments were performed in either duplicate or triplicate and repeated five times. The *in vivo* experiments were normalised to controls and data was expressed as percentage control. *p* < 0.05 was considered significant. All statistical analyses were performed using GraphPad Prism 9.3.1 (GraphPad Software, LLC).

## 3 Results

### 3.1 Placental galectin-3 is reduced in the patients with early-onset preeclampsia

**FIGURE 1:**
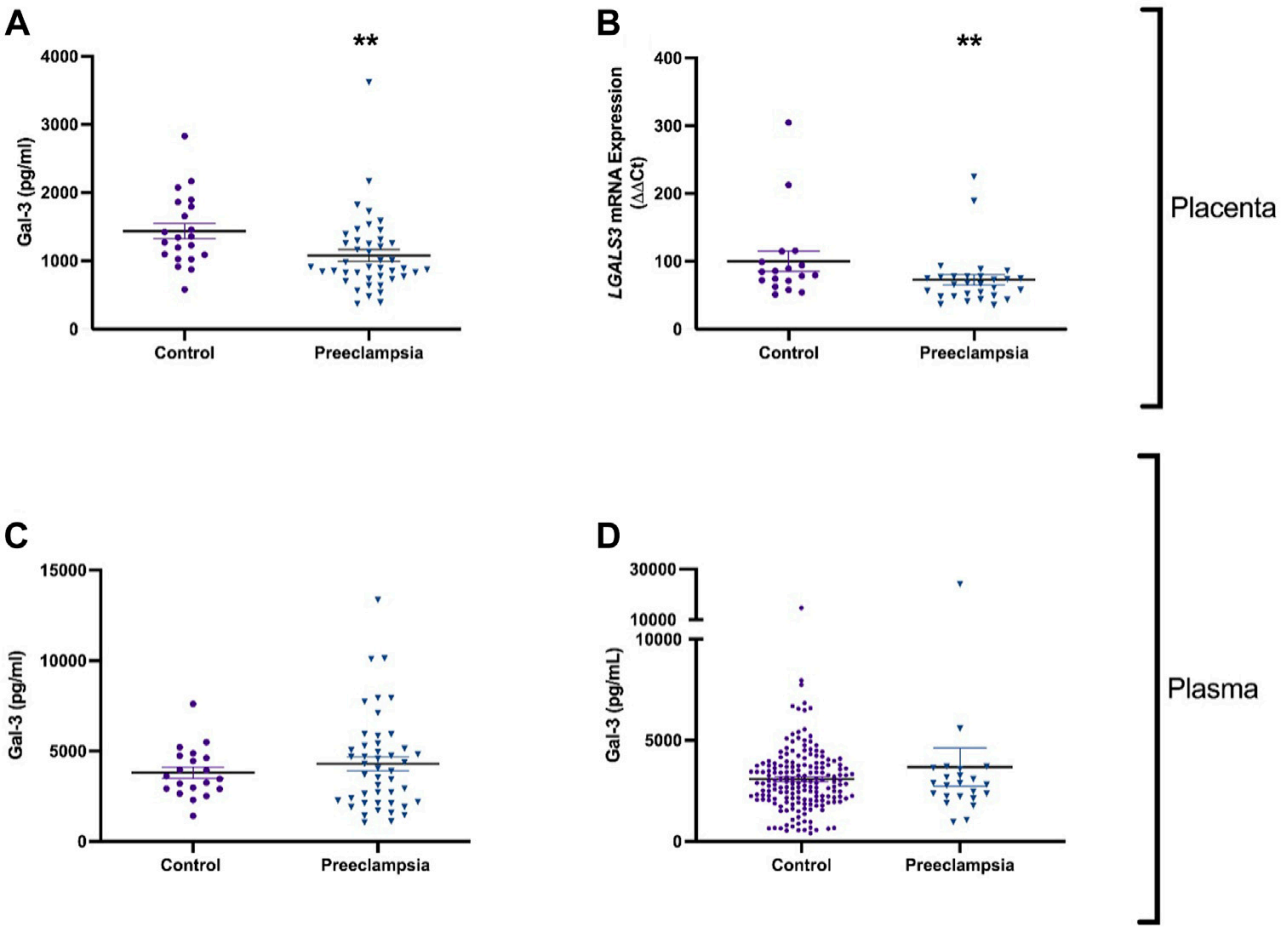
Galectin-3 is reduced in the placenta, but not in plasma of patients with early-onset preeclampsia or before the diagnosis of preeclampsia. Galectin-3 protein concentration in placental lysates from 43 patients with early-onset preeclampsia and 21 controls **(A)**. mRNA expression of galectin-3 gene *LGALS3* in placenta from 29 women with preeclampsia and 18 controls **(B)**. Circulating Galectin-3 in 46 patients with early-onset preeclampsia and 20 controls **(C)**. Galectin protein in maternal plasma from 23 women who later developed preeclampsia, and 182 healthy controls **(D)**. Data points represents individual patients (control; purple and preeclampsia; blue). Data are expressed as median (Interquartile range). **p < 0.01.

Galectin-3 protein and mRNA expression were measured in placenta of patients with early-onset preeclampsia who delivered before 34 weeks’ gestation. Galectin-3 protein was significantly decreased in placenta from pregnancies (*p* = 0.002) complicated by preeclampsia (*n* = 43) compared to gestation matched controls (Figure 1A; *n* = 21). mRNA expression of LGALS3 was significantly reduced (*p* = 0.009) in placentas from women with preeclampsia (*n* = 29) compared to controls (Figure 1B; *n* = 18).

### 3.2 Circulating galectin-3 in established early-onset preeclampsia and preceding a diagnosis ofpreeclampsia at term gestation

Given that galectin-3 protein and expression levels were dysregulated in preeclamptic placental lysates, we measured circulating galectin-3 in patients with established early-onset disease relative to gestation matched controls and at 36 weeks prior to any potential term preeclampsia diagnosis. There was no significant difference in circulating galectin-3 levels in women with early-onset preeclampsia (*n* = 46) compared to controls (*n* = 20, Figure 1C). Galectin-3 levels were not different in women who later developed preeclampsia (*n* = 23) versus controls (*n* = 182) (Figure 1D).

### 3.3 *LGAL*S3 and LGALS3BP mRNA expression in differentiated first trimester cytotrophoblast into syncytiotrophoblast or extravillous trophoblast cells

**FIGURE 2:**
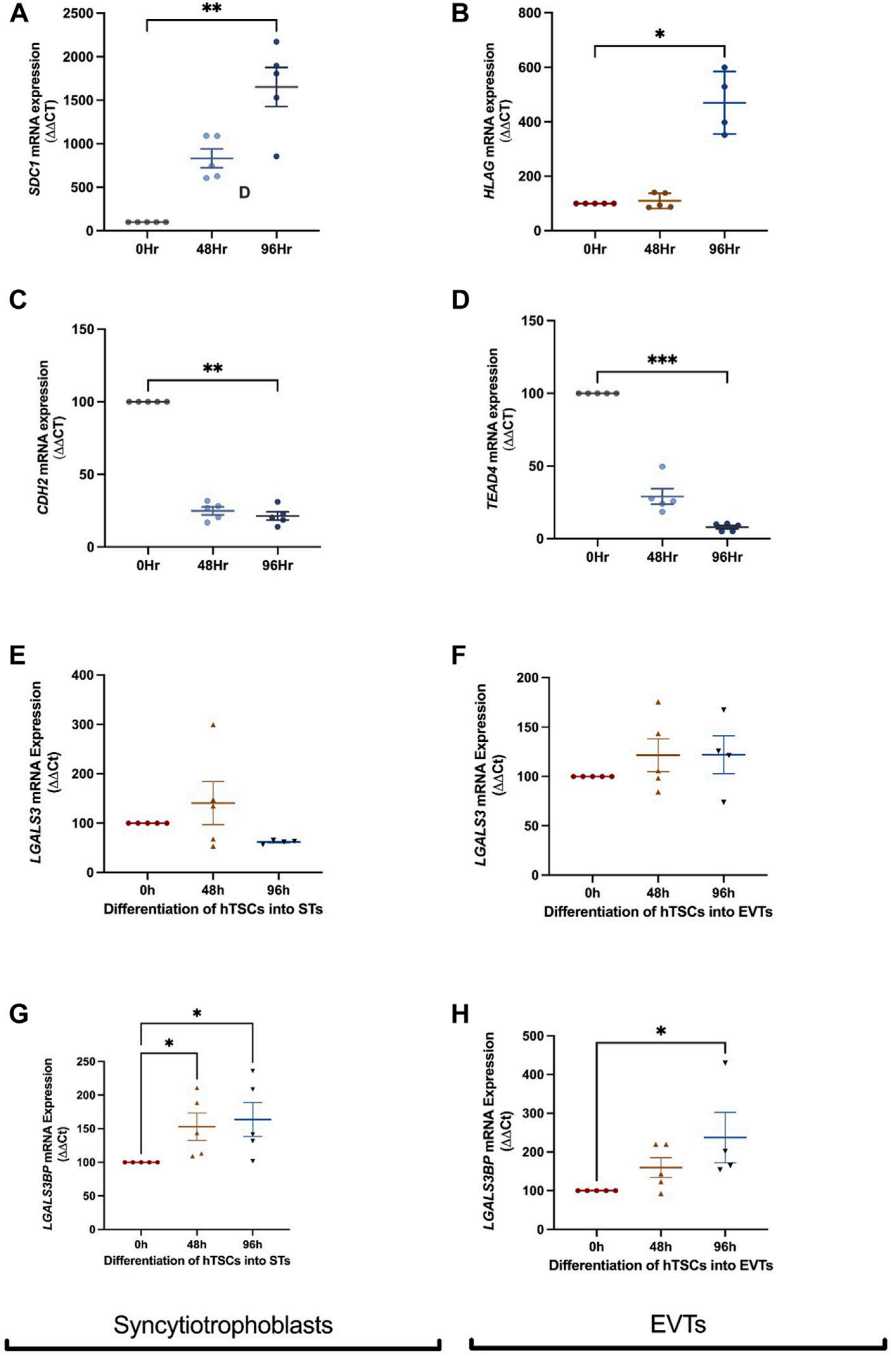
LGALS3 and LGALS3BP mRNA expression in first trimester placental stem cells differentiated into syncytiotrophoblast and extravillous trophoblasts. First trimester placental cytotrophoblast cells were differentiated into either syncytiotrophoblast or extravillous trophoblast (EVT) cells over 96 h. Syncytiotrophoblast differentiation was confirmed by increased expression of *SDC1* (syncytiotrophoblast marker) **(A)** and decreased expression of *CDH2* (cell border marker) **(C)** across time. LGALS3 **(E)** and LGALS3BP **(G)** mRNA expression with syncytiotrophoblast differentiation across 96 h. EVT differentiation was confirmed by increased expression of HLAG (EVT marker) **(B)** and reduced expression of TEAD4 (cytotrophoblast marker) **(D)** across time. LGALS3 **(F)** and LGALS3BP **(H)** mRNA expression with EVT differentiation over 96 h. All experiments were repeated *n* = 5 times in duplicate. Data is expressed as mean ± SEM; *p < 0.05, **p < 0.01.

To characterise the expression of galectin-3 and galectin-3BP in the placenta, cytotrophoblast were differentiated into either syncytiotrophoblast or EVT across 96h, and mRNA was measured at 0, 48, and 96 h time points. Syncytialisation was confirmed by increased SDC1 (syncytiotrophoblast marker) expression (Figure 2A, *p* = 0.001) and reduction in cell border marker, CDH2 (E-cadherin 2) mRNA expression with syncytiotrophoblast differentiation over 48 and 96 h (Figure 2C, *p* = 0.005). In addition, LGALS3 mRNA expression did not change as cytotrophoblast cells were differentiation into syncytiotrophoblast cells (Figure 2E). However, *LGALS3BP*mRNA expression was increased following differentiation into syncytiotrophoblast cells (Figure 2G, *p* = 0.01).

Differentiation of hTSCs into EVTs was confirmed by an increase in HLAG (EVT marker) expression after 96 h (Figure 2B, *p* = 0.03) and reduction in cytotrophoblast marker TEAD4 mRNA expression after 96 h (Figure 2D, *p* = 0.0006). There were no differences in LGALS3 mRNA expression following cytotrophoblast differentiation into EVT cells (Figure 2F). However, LGALS3BP mRNA expression was significantly increased (Figure 2H, *p* = 0.02) following EVT differentiation.

### 3.4 *LGALS3* and *LGALS3BP* mRNA expression in first trimester placental stem cells exposed to either hypoxia or inflammatory stimuli

**FIGURE 3:**
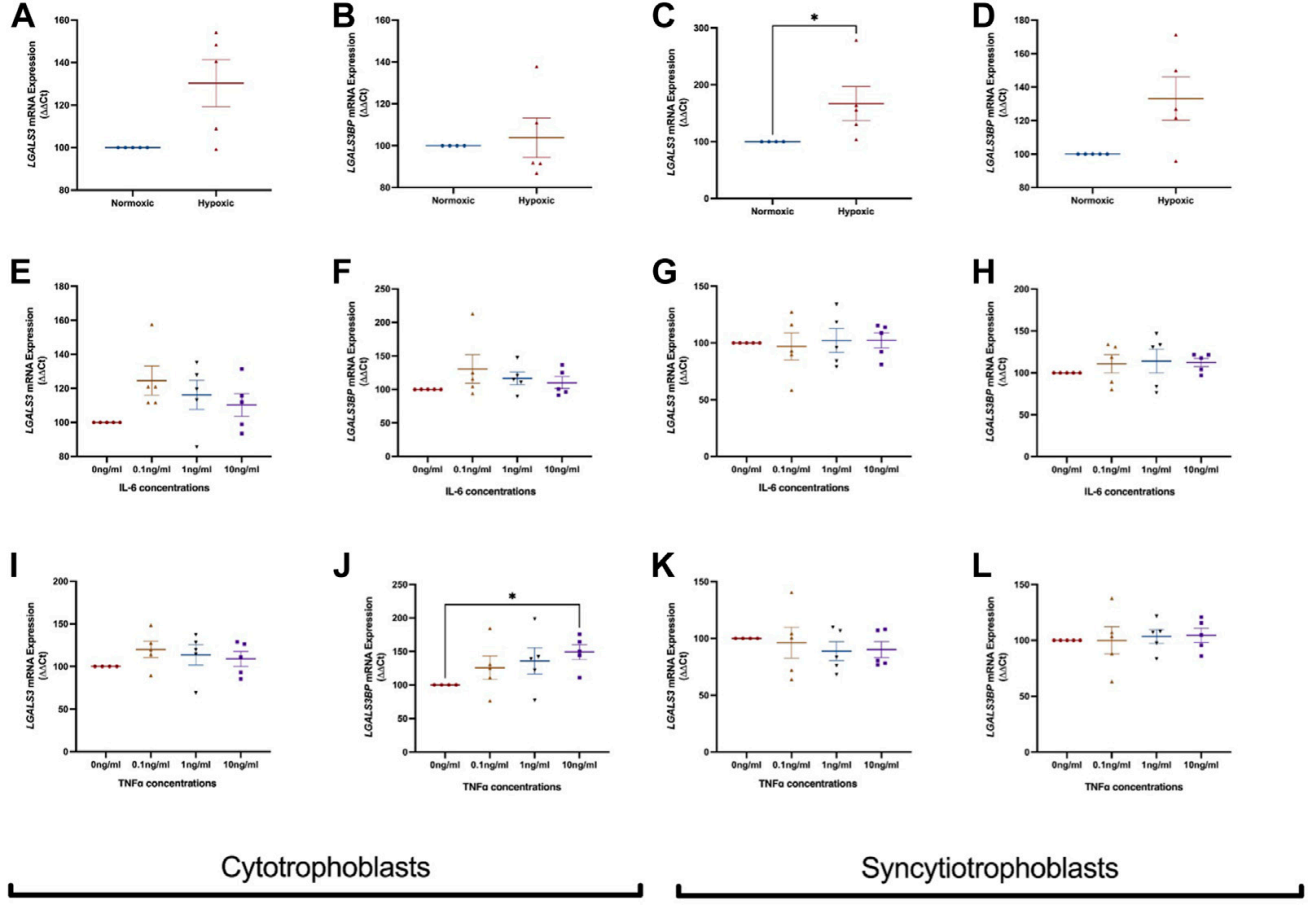
Effect of hypoxia and inflammation on LGALS3 and LGALS3BP mRNA expression in cytotrophoblast and syncytiotrophoblast cells. First trimester cytotrophoblast and syncytiotrophoblast cells were cultured in hypoxia or exposed to inflammatory stimuli (IL-6 or TNFα) at 0, 0.1, 1, or 10 ng/ml. mRNA expression of LGALS3 **(A)** and LGALS3BP **(B)** in cytotrophoblast cells after 1% hypoxia or 8% normoxia exposure. mRNA expression of LGALS3 **(C)** and LGALS3BP **(D)** in syncytiotrophoblast cells following 1% hypoxia and 8% normoxia conditions. LGALS3 **(E)** and LGALS3BP **(F)** mRNA expression following IL-6 treatment in cytotrophoblast cells. LGALS3 **(G)** and LGALS3BP **(H)** mRNA expression following IL-6 treatment in syncytiotrophoblast cells. LGALS3 **(I)** and LGALS3BP **(J)** mRNA expression in cytotrophoblast cells following TNFα treatment. LGALS3 **(K)** and LGALS3BP **(L)** mRNA expression in syncytiotrophoblast cells following TNFα treatment. All experiments were repeated *n* = 5 times with triplicate repeats. Data is expressed as mean ± SEM; *p < 0.05.

Next, LGALS3 and LGALS3BP mRNA expression was measured in cytotrophoblast and syncytiotrophoblast cells exposed to hypoxia (1% O2) or normoxia (8% O2). In cytotrophoblast cells, LGALS3 (Figure 3A) and LGALS3BP (Figure 3B) mRNA expression was unchanged in a hypoxic environment. Hypoxia increased mRNA expression of LGALS3 in syncytiotrophoblast cells (Figure 3C, *p* = 0.016), but not LGALS3BP expression (Figure 3D).

We exposed cytotrophoblasts and syncytiotrophoblast to pro-inflammatory cytokines IL-6 and TNFα to determine if inflammation dysregulates *LGALS3* and LGALS3BP expression. Treatment of IL-6 did not alter mRNA expression of *LGALS3* (Figure 3E) and LGALS3BP (Figure 3F) in cytotrophoblast cells. mRNA expression of *LGALS3* (Figure 3G) and LGALS3BP (Figure 3H) was also not changed in syncytiotrophoblast cells.

When cytotrophoblast (Figure 3I) and syncytiotrophoblast (Figure 3K) cells were treated with TNFα, no change in *LGALS3* mRNA expression was observed. However, there was increased LGALS3BP expression in the presence of TNFα in cytotrophoblast cells (Figure 3J, *p* = 0.03). TNFα did not alter LGALS3BP expression in syncytiotrophoblast (Figure 3L).

## 4 Discussion

This study identified reduced levels of placental galectin-3 in women with early-onset preeclampsia but no changes within the circulation in established early-onset disease or before development of preeclampsia. The cell studies revealed increased LGALS3 expression in hypoxia treated syncytiotrophoblast cells and LGALS3BP expression in TNF-α treated cytotrophoblast cells.

Galectin-3 is a carbohydrate binding lectin that plays a crucial role in many diseases (Simovic Markovic et al., 2016). Our study revealed reduced placental galectin-3 in established early-onset preeclampsia (delivered at <34 weeks’ gestation). In a mouse model of pregnancy with galectin-3 knockdown, reduction in galectin-3 was accompanied by reduced fetal weight, delay in fetal development, and increased placental inflammation (Freitag et al., 2020). Although our study did not detect changes in galectin-3 in plasma from women with preeclampsia, a reason for this may be due to a reduction of galectin-3 production in the placenta, which reduces the amount secreted into the maternal circulation. In contrast to our findings, Ruikar et al. (2022) and others have demonstrated elevated levels of galectin-3 protein in placentas from preeclamptic pregnancies (Jeschke et al., 2007). A possible reason for the discrepancy in findings is Ruikar et al. (2022) measured galectin-3 in preterm preeclamptic placentas, compared with term controls, whilst our study looked at early-onset preeclamptic placentas compared to gestation matched controls. Several studies have suggested differences in the aetiology of early-onset and term preeclampsia, therefore the underlying abnormalities contributing to early-onset preeclampsia may not be comparable to term disease (Gathiram and Moodley, 2016; Phillips et al., 2010). Further studies are required to validate these findings and determine how placental galectin-3 protein differs in early-onset and late-onset preeclampsia in both phenotypes.

This study is the first to evaluate circulating galectin-3 in plasma from women with preeclampsia. In our established disease cohort, circulating plasma galectin-3 was not altered in women with early-onset preeclampsia. Other studies have measured galectin-3 in serum. A recent study measured serum galectin-3 in early-onset preeclampsia also found no significant differences between preeclampsia and controls (Nikolov et al., 2020). However, Pankiewicz et al. (2020) reported higher serum galectin-3 levels in patients with preeclampsia. It is important to note that their study involved samples from both early-onset and late-onset preeclampsia, while our established disease cohort all delivered early-onset (<34 weeks).

In total, there are at least 15 known galectins, and other studies have examined many of them in reproductive tissues from healthy and pathological pregnancies. Galectin-1 likely overlaps with galectin-3 as both are reported to increase with trophoblast invasion and syncytilisation (Jeschke et al., 2007). Although galectin-1 differs from galectin-3 through support of immune tolerance and by influencing the secretion of hCG (Blois and Barrientos, 2014). Galectin-1 is increased in the placenta of patients diagnosed with severe preeclampsia compared to gestation matched controls (Than et al., 2008). As galectin-1 mediates a variety of immune cell interactions and responds to acute inflammation (Rabinovich et al., 2000), the increased expression could be part of a placental response to increased maternal inflammation, which could influence the maternal-fetal tolerance. Placental specific galectin-13 is a well-studied protein that has a role in damage signalling as it is elevated with the onset of preeclampsia. As galectins can be secreted from inflamed tissues following cellular stress, future research would benefit from measuring multiple galectins with galectin-3 to understand their diverse roles in placentas complicated by preeclampsia.

There is limited literature to suggest that LGALS3BP is expressed in all trophoblast subpopulations. Our *in vitro* differentiation studies identified higher LGALS3BP expression in syncytiotrophoblast and EVT compared to cytotrophoblast cells. mRNA expression of LGALS3 was not altered in cytotrophoblast, syncytiotrophoblast or EVT. While the binding protein is only increased in syncytiotrophoblast and EVTs, all trophoblast cell types can produce the ligand galectin-3. Several studies have reported the interaction between galectin-3 and galectin-3 binding protein initiates pathologic, proinflammatory signalling cascades in diseases such as cancer, and venous thrombosis (DeRoo et al., 2015; Newlaczyl and Yu, 2011; Silverman et al., 2012). A study conducted by Silverman et al. reported that interaction between galectin-3 and galectin-3 binding protein resulted in transcriptional upregulation of IL-6 in bone marrow mesenchymal stem cells *via* galectin-3 binding protein/galectin-3/Ras/mEK/ERK signalling pathway (Silverman et al., 2012). Given inflammation and placental hypoxia play crucial role in pathophysiology of preeclampsia (Roberts and Gammill, 2005), we examined the effect of pro-inflammatory cytokines (IL-6 and TNFα) and hypoxia on LGALS3 and LGALS3BP mRNA expression in placental cytotrophoblast and syncytiotrophoblast cells. In our study, treatment of pro-inflammatory cytokine TNF-α caused an increase in LGALS3BP expression in cytotrophoblast, whilst IL-6 had no effect on LGALS3BP mRNA expression. A study by Gleissner et al. (2017) measured galectin-3BP concentrations in plasma from patients with cardiovascular disease and found increased galectin-3BP were associated with enhanced markers of inflammation, including TNFα. This study shares similarities to ours as we have observed increased LGALS3BP mRNA expression with increased inflammation, indicating the possible involvement of galectin-3BP in inflammation associated with preeclampsia. In addition, previous proteomic analysis by Kolla et al. (2012) reports elevated levels of circulating galectin-3 binding protein in patients at high risk of developing preeclampsia. Given galectin-3 binding protein binds to other galectins and there are other galectins expressed in the fetal-maternal interface (Jovanović Krivokuća et al., 2021), further studies should investigate the role of galectin-3 binding protein in the presence of other galectins in preeclampsia.

Previous studies have reported increased galectin-3 levels in human placenta cell line from choriocarcinoma BeWo cells when cultured in hypoxic conditions (Hu et al., 2007). Our data also suggests that the galectin-3 gene is increased with hypoxia. This is different to our data in preeclamptic placentas where we found decreased galectin-3 protein and mRNA expression. Therefore, the dysregulated galectin-3 that was observed in early-onset preeclampsia is unlikely a result of hypoxia.

Collectively, this study observed dysregulated levels of galectin-3 in early-onset preeclamptic placental lysates and hypoxia, but not inflammation. While results suggest a potential association between galectin-3 binding protein and inflammation, further studies are needed to understand the relationship between galectin-3 binding protein and galectin-3 in preeclampsia.

## Data Availability

The raw data supporting the conclusion of this article will be made available by the authors, without undue reservation.
